# Atmospheric Cold Plasma Inactivation of *Salmonella* and *Escherichia coli* on the Surface of Golden Delicious Apples

**DOI:** 10.3389/fnut.2018.00120

**Published:** 2018-12-11

**Authors:** Agnes Kilonzo-Nthenge, Siqin Liu, Sudheer Yannam, Ankit Patras

**Affiliations:** ^1^Department of Human Sciences, Tennessee State University, Nashville, TN, United States; ^2^Department of Agricultural and Environmental Sciences, College of Agriculture, Tennessee State University, Nashville, TN, United States

**Keywords:** atmospheric cold plasma, *Salmonella*, *Escherichia coli*, apples, modeling

## Abstract

The contamination of fruits with human pathogens is a reoccurring concern in the fresh produce industry. Atmospheric cold plasma (ACP) is a potential alternate to customary approaches for non-thermal decontamination of foods. In this study, the efficacy of a dielectric barrier discharge ACP system against *Salmonella* (*Salmonella* Typhimurium, ATCC 13311; *Salmonella* Choleraesuis, ATCC 10708) and *Escherichia coli* (ATCC 25922, ATCC 11775) was explored. For each bacteria, a two-strain mixture at 8 log_10_ CFU/ml was spot inoculated on the surface of Golden Delicious apples, air dried, and exposed to ACP at a fixed distance of 35 mm, input power of 200 W for 30, 60, 120, 180, and 240 s. Bacterial inactivation was achieved in all treatment times with highest reduction of 5.3 log_10_ CFU/cm^2^ for *Salmonella* and 5.5 log_10_ CFU/cm^2^ for *E. coli*. Our results showed that reductions were interrelated to exposure time and ranged from 1.3 to 5.3 and 0.6 to 5.5 log_10_ CFU/cm^2^ for *Salmonella* and *E. coli*, respectively. *Salmonella* and *E. coli* significantly decreased (>5.0 log) at 180 and 240 s as compared to 30, 60, and 120 s exposure. Microbial inactivation data was modeled by using Weibull distribution. These findings demonstrate the potential of ACP as a postharvest technology to effectively reduce pathogens on apples, with reference to *Salmonella* and *E. coli*.

## Introduction

Consumer demand for nutritious, safe, and minimally treated foods has stimulated the increased consumption of fresh produce (1). Several studies reveal that consuming more fruits and vegetables result to a more prolific and healthier lifestyle (2, 3). However, the number of foodborne illness outbreaks linked to fresh produce has elevated in the recent years. Between November 2010 and November 2012, 5191 individuals were infected with foodborne pathogens from fresh produce products and as a consequence 95 people died in European countries, USA, Canada, and Japan (4, 5). According to Painter et al. (6), 46% of foodborne illnesses are associated with fresh produce. Fresh produce is recognized as a highly potential vehicle for foodborne outbreaks and therefore, a major concern to the food industry, regulatory agencies, and consumers (7, 8). The United States and European Union have reported a total of 377 and 198 produce-associated outbreaks from year 2004 to 2012. For the United States, the absolute number of outbreaks due to fresh produce ranged from 23 to 60 per year. There were substantial increases in 2006 (57 outbreaks), 2008 (51 outbreaks), and 2011 (60 outbreaks) (9, 10). For the European Union, the number of outbreaks oscillated between 10 and 42, highlighting increases in 2006 (29 outbreaks), 2009 (34 outbreaks), and 2010 (44 outbreaks) (11). The number of produce-associated outbreaks remains relatively high and represents a major health and financial issue (11, 12).

Consumer's demand for safe, high quality and wholesome food has contributed to the development of novel non-thermal technologies (13). The preservation of food quality is of paramount importance, and practice application must be effective at destroying microorganisms while not causing undesirable alterations in food quality (7). Atmospheric cold plasma (ACP) is a fairly new technology being applied for non-thermal decontamination of foods. This method promotes a proficient inactivation of diverse microorganisms including spores, viruses, yeasts, and fungi including biofilms (14–23).

Plasma involves very energetic species including photons, electrons, positive and negative ions, free radicals and excited or non-excited molecules and atoms, which in combination inactivate microorganisms (24–26). Cold atmospheric plasma, grounded on ionized gases generated at room temperature and atmospheric pressure presents the opportunity of treating the surfaces of fresh produce tissues (27–29). Various types of plasma generating sources are currently available. It includes glow-discharge, radio-frequency discharge, corona discharge, dielectric barrier discharge, atmospheric pressure plasma jet, micro-hollow, gliding arc discharge have been used for food processing applications, generating plasmas by using noble gases is more beneficial as it prevents the oxidative degradation of vitamins and other nutrients on exposure to oxygen. The most economical operations in food systems employ atmospheric air (14, 30) which is relatively cheaper.

Vegetative cells such as *E.coli* O157:H7 and *Salmonella* are often associated with fresh produce, hence remains to be a threat to the public health (2, 31). New technologies such as gas plasma are needed to inactivate pathogens and prevent cross-contamination. Extensive research on the use of plasmas at atmospheric pressure using air as a carrier gas to inactivate microorganisms is a relatively recent phenomenon. Atmospheric pressure cold plasma is an emerging low temperature technology with high antimicrobial efficacy. Gas plasma technique is claimed to be a “rapid, waterless, zero-contact, chemical-free” tool for pathogen removal from food contact surfaces (14, 20).

Several authors reported non-thermal atmospheric plasma treatments for decontamination of many food samples including vegetables, meat and meat products, milk and dairy products, fruit juice (32–34). This study fills that knowledge gap and provides inactivation data on a range of vegetative cells. Not much information on the effect of plasma using air as a carrier gas on fruits is available. Therefore, using purified air as a carrier gas for the plasma generation is the novelty of this research study. The main objective of this study was to evaluate the efficacy of atmospheric cold plasma in reducing *Salmonella* and *Escherichia* coli on the surface of Golden Delicious apples. A further aim was to evaluate the kinetic models for the inactivation of *Salmonella* and *Escherichia* coli on apples.

## Materials and Methods

### Bacterial Strain and Inoculation Preparation

*Salmonella enterica* subspecies *enterica* serovars (Typhimurium, Choleraesuis) and *Escherichia coli* (*E. coli* ATCC 25922; *E. coli* ATCC 11775) were used in this study. Isolates were maintained at −80°C in tryptic soy broth (TSB; Becton, Dickinson & Company [BD], Franklin Lakes, NJ) with 15% glycerol (Fisher Scientific, Pittsburgh, PA). Culture from each bacterial frozen stock was separately inoculated on tryptone soya agar (TSA) plates and grown overnight at 37°C to isolate pure colonies. An isolated colony from each plate was transferred to corresponding 10 TSB tube and incubated with shaking (250 rpm) at 37°C to create working stock. Subsequently, bacterial cells from each tube were harvested through centrifugation (4,000 × g, 15 min, 23°C) and suspended in 10 ml of 0.1% peptone water. Approximately, 5 ml of each resuspended *Salmonella enterica* subspecies *enterica* serovars (Typhimurium, Choleraesuis) cells were combined to make a 10 ml cocktail (a two-strain cocktail mixture). The same procedure used to make *Salmonella* cocktail was followed for *E. coli* cocktail *(E. coli* ATCC 25922; *E. coli* ATCC 11775). Separately, 1 ml of each bacterial cocktail was re-suspended in 9 ml 0.1% peptone and there after serial dilutions mere performed. Next, 0.1 ml from each cocktail was plated on TSA plates and a final inoculum was determined as approximately 8 log_10_ CFU/ml for both *Salmonella* and *E. coli*.

### Inoculation of Golden Delicious Apples

The efficacy of ACP on *Salmonella* and *E. coli* was evaluated using apples as the test substrate. Fresh Golden Delicious apples were randomly picked and purchased from local supermarkets and held at 4°C 1 day before the experiment. Prior to inoculating the apples with *Salmonella* or *E. coli* cocktail, each apple was dipped in 70% ethanol for 10 s, rinsed with distilled sterile water, and allowed to air dry for 1 h in a safety hood cabinet. For negative control (before inoculation), six apples was analyzed to be certain there was no baseline contamination of *Salmonella* or *E. coli* on the apples. The apples were cut in halves and placed cut side down onto sterile petri plates. To facilitate inoculation process, a sharpie was used to denote the area of inoculation (12.6 cm^2^) on the apple halves (29). Next, 0.1 ml (100 μl) aliquots of *E.coli* or *Salmonella* cocktail were spot inoculated on the denoted area. The inoculum was deposited in form of droplets on several different sites within the denoted area, to ensure that the inoculum did not flow to the side of the apples. After inoculation, samples were then left to dry for 1 h in a laminar flow safety cabinet to allow the attachment of bacteria on the surface of apples prior to the ACP treatment (35). Following, inoculated apples were treated by ACP and analyzed for microbial inactivation. Four apples were used for each set of treatment and the entire study was accomplished in triplicates.

### Indirect Corona Discharge Set-Up by Atmospheric Cold Plasma Treatment

**Figure 1:**
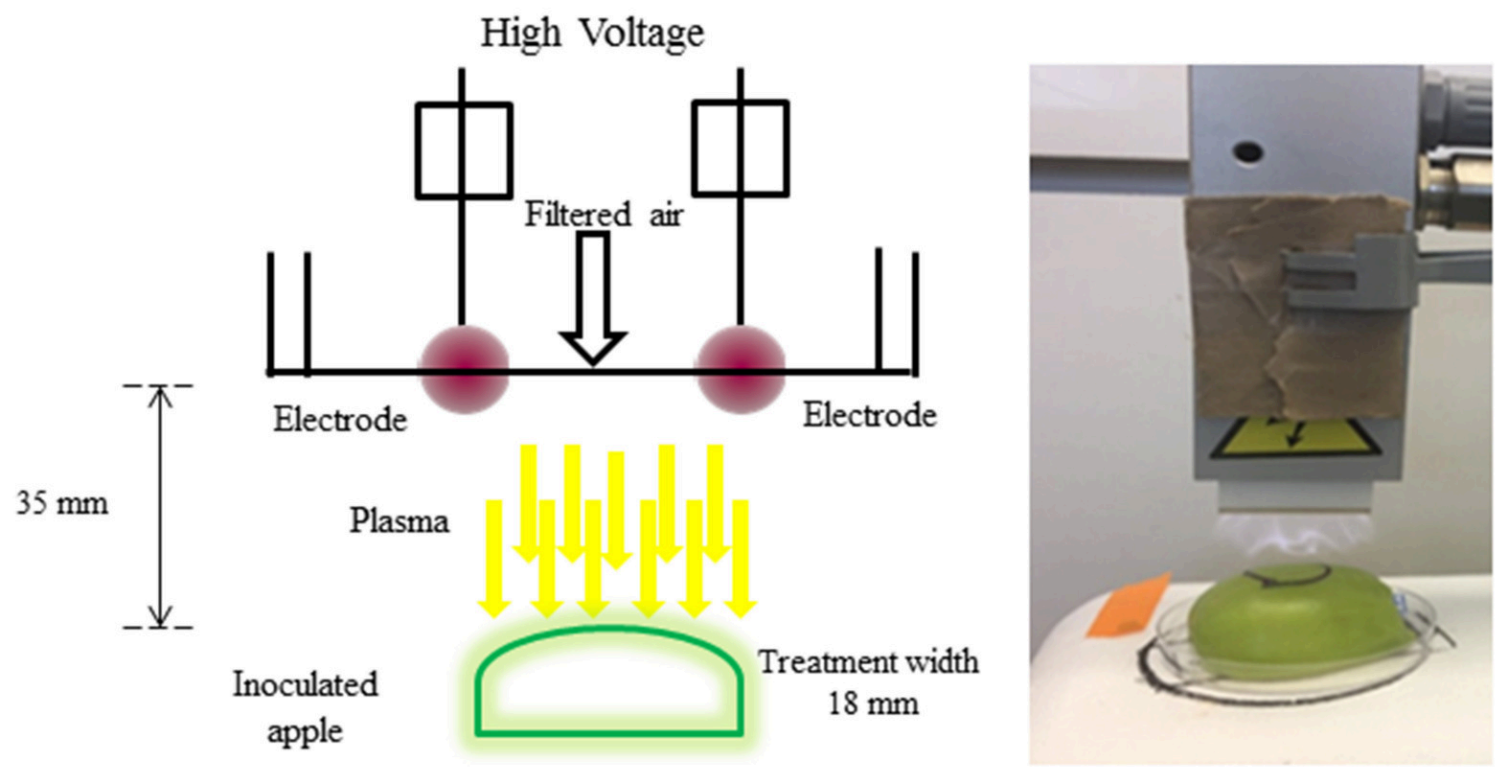
Schematic diagram of atmospheric cold plasma system.

To treat the apples with ACP, indirect corona discharge system was used in this study (Figure 1). It consisted of a maximum high voltage output of 200 Watts at a frequency of 50 Hz. The Corona T-JET enables an indirect Corona-treatment with very low heat transfer into the surface. The corona discharge was generated inside the head between two electrodes and conveyed onto the surface by an air stream. A corona discharge is a physical phenomenon characterized by a low-current electrical discharge across a gas containing gap at a voltage gradient, which exceeds a certain critical value. Filtered air at a pressure of 4 bar at a constant flow-rate of 17 L min^−1^ was used for the plasma generation. The process parameters considered for the treatment was plasma at a fixed distance of 35 mm. Treatment width was approximately 18 mm (Figure 1). All samples were subjected to plasma treatment under atmospheric pressure. The plasma working gas was atmospheric air. The samples were treated with plasma at intervals of 30, 60, 120, 180, and 240 s. A mark was made on the surface to denote the orientation of the apple with respect to the rectangular field of plasma. The chemical characterization of the emission in the 220–450 nm wavelength range (Figure 1) was carried out by an optic fiber probe placed at about 20 mm from the discharge and connected to a spectrometer (Ocean optics, HR4000 Series, Florida, USA).

### Microbial Analysis

*E. coli* and *Salmonella* contaminated apples (positive control) were processed individually; this was to determine the inoculum levels before ACP treatment. After ACP treatment, the inoculated spot (12.6 cm^2^) on the apple was sampled using a sterile cotton swab immersed in sterile 0.1% buffered peptone water. The cotton swab tip was thoroughly vortexed for 30 s in 10 mL 0.1% (w/v) sterile peptone in conical tube. Subsequently, a 10-fold serially dilutions in 0.1% peptone of the homogenate from each sample was plated on appropriate media. Approximately, 0.1 ml aliquots of an appropriate dilution were surface plated on xylose lysine deoxycholate (XLD, Difco, Sparks, MD) agar and Eosin Methylene Blue Lactose Agar (EMB) for *Salmonella* and *E. coli*, respectively. All plates were incubated for 24 h at 37°C.

### Inactivation Kinetics

The GInaFiT software tool was used to perform the regression analysis of the microbial inactivation data (36). To describe the survival of *S*. Typhimurium and *E. coli*, different inactivation models are available in the GInaFiT tool, namely the log-linear, log-linear +shoulder, Weibull, Double Weibull, biphasic and biphasic+ shoulder models (Van Boekel 2). Weibull model fitted the experimental data. Inactivation kinetics parameters related to scale and shape of inactivation curves for the model was calculated. The numerical values for inactivation kinetics parameters, time required to obtain 5-log reduction and other similar parameters were calculated for each model. It is well-known that microbial activation curves are not often straight lines but have a “shoulder” or “tail” effect; thus, the Weibull distribution model was preferred to fit microbial inactivation data (36, 37). The Weibull model has been used in non-thermal treatment studies for modeling purposes. It is primarily based on the hypothesis that the resistance to stress of a population follows a Weibull distribution (38, 39). The Weibull model (eqn 1) was used to analyze the data: where N_t_ is concentration of microorganisms (CFU/cm^2^) at time, t (0, 30, 60, 120, 180, 240 s), N_0_ (CFU/cm^2^) is the initial number of microorganisms, δ [min] (time for the first decimal reduction) and p [–] are parameters related to the scale and shape of the inactivation curve, respectively. The Weibull distribution corresponds to a concave upward survival curve if *p* < 1 and concave downward if *p* > 1 (40). Inactivation kinetics parameters related to scale and shape of inactivation curves for most suitable models were calculated. The numerical values for inactivation kinetics parameters, time required to obtain 5-log reduction were calculated for the model. The values of δ and *p* were used to calculate a desired log reduction. The time required to obtain an x log reduction (t_xd_) was calculated using Equation (2).

β describes the shape of the curve (β = 1, straight line; β < 1, concave curve; β > 1, convex curve). The parameter α modifies the slope but it does not affect the shape (40). The Weibull equation can be cast in the decimal logarithmic form.

(1) $$\begin{array}{r} Log_{10}N_{\text{t}}=Log_{10}\left(N_{0}\right)-{\left(\frac{t}{\delta }\right)}^{p} \end{array}$$

(2) $$\begin{array}{r} t_{\text{xd}}=\delta \times {\left(x\right)}^{1/\text{p}} \end{array}$$

### Statistical Analysis

Statistical Analysis (SAS Institute, Cary, N.C.) program was used to analyze the data. The surviving population of either *E. coli* or *Salmonella* for each treatment was compared with that recovered from its respective inoculated untreated spot (control). This was performed to account for possible day-to-day variation in inoculum strength over the course of the experiments. All plate count data were converted to log CFU/cm^2^ values. A balanced design with three replicates randomized in experimental order were performed for each treatment. The concentrations of *E. coli* and *Salmonella* spp. (Log CFU/cm^2^) after plasma treatment were analyzed in the Weibull frequency distribution model. We used an independent set of data to validate the models. Consequently, based on the validation statistics obtained from using an independent set of experimental data, the predictive results from the above model can be considered accurate. *P*-values > 0.05 were considered statistically significant.

Our results showed that both the plasma exposure time as well as the fixed gas composition played a key role regarding the inactivation of *E. coli* and *Salmonella* on apples. Showing dependence on the plasma exposure time, downward concave survival curves were observed. The parameters for Weibull models are shown in Table 1. The goodness-of-fit of the inactivation models was compared by determining the *R*^2^ values. The goodness-of-fit of the models is described by the root mean square error (RMSE), which was between 0.17 and 0.53 and which thus proved the suitability of the Weibull model.

**Table 1:** Goodness of fit and model parameters.

**Microbe**	***R*^**2**^**	**RMSE**	**δ ± SE**	**P ± SE**
*Salmonella* Typhimurium *ATCC 13311*	0.92	0.48	12.73 ± 4.78	0.52 ± 0.06
*Salmonella* Choleraesuis *ATCC 10708*	0.94	0.53	22.02 ± 5.65	0.80 ± 0.1
*Escherichia coli* ATCC 25922	0.96	0.38	12.26 ± 2.87	0.59 ± 0.04
*Escherichia coli* ATCC 11775	0.99	0.17	46.70 ± 2.84	1.20 ± 0.05

*SE, Standard error; RMSE, Root mean square error*.

## Results and Discussion

### Inactivation of Bacteria on Golden Delicious Apples

**Figure 2:**
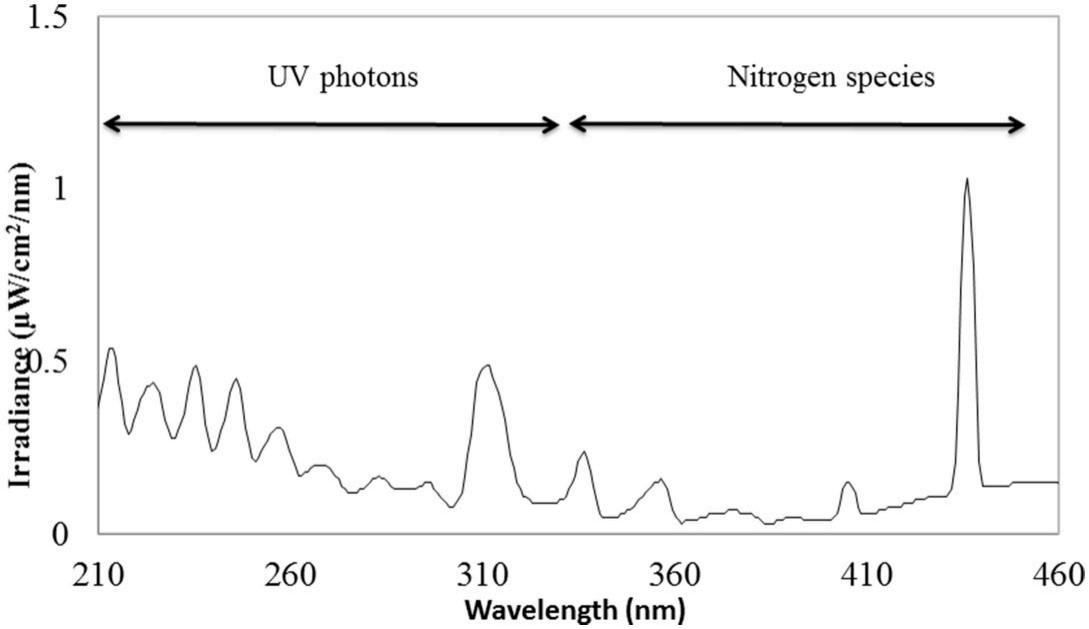
Optical emission spectrum of plasma.

The schematic view of the ACP unit is shown in Figure 1. Emission spectra from the plasma discharge is shown in Figure 2. It is quite evident from the spectra (Figure 2), the major emission lines were in the 300–450 nm regions and attributed to nitrogen species. These bands are associated with the transition from the second positive system of N_2_, the first negative system of ${\text{N}}_{2}^{+}$and the beta and gammas system of NO. Moreover, the lines between 315 and 405 nm are linked to the second positive system of N_2_ whilst the bands approximately at 391 and 426.5 nm are due to the first negative system of ${\text{N}}_{2}^{+}$(41). It was observed that a strong emission peak in the UV range was also observed which could be attributed to UV photons in the germicidal range.

The influence of ACP treatments on viability of *Salmonella* and *E. coli* was investigated in our study. Atmospheric plasma, a non-thermal surface decontamination technique is capable of generating various groups of antimicrobial agents which includes photons, electrons, positively and negatively charged ions, atoms, free radicals and excited or non-excited molecules (42, 43). In our study, plasma discharge consisted of nitrogen species and UV photons as illustrated in Figure 2.

**Figure 3:**
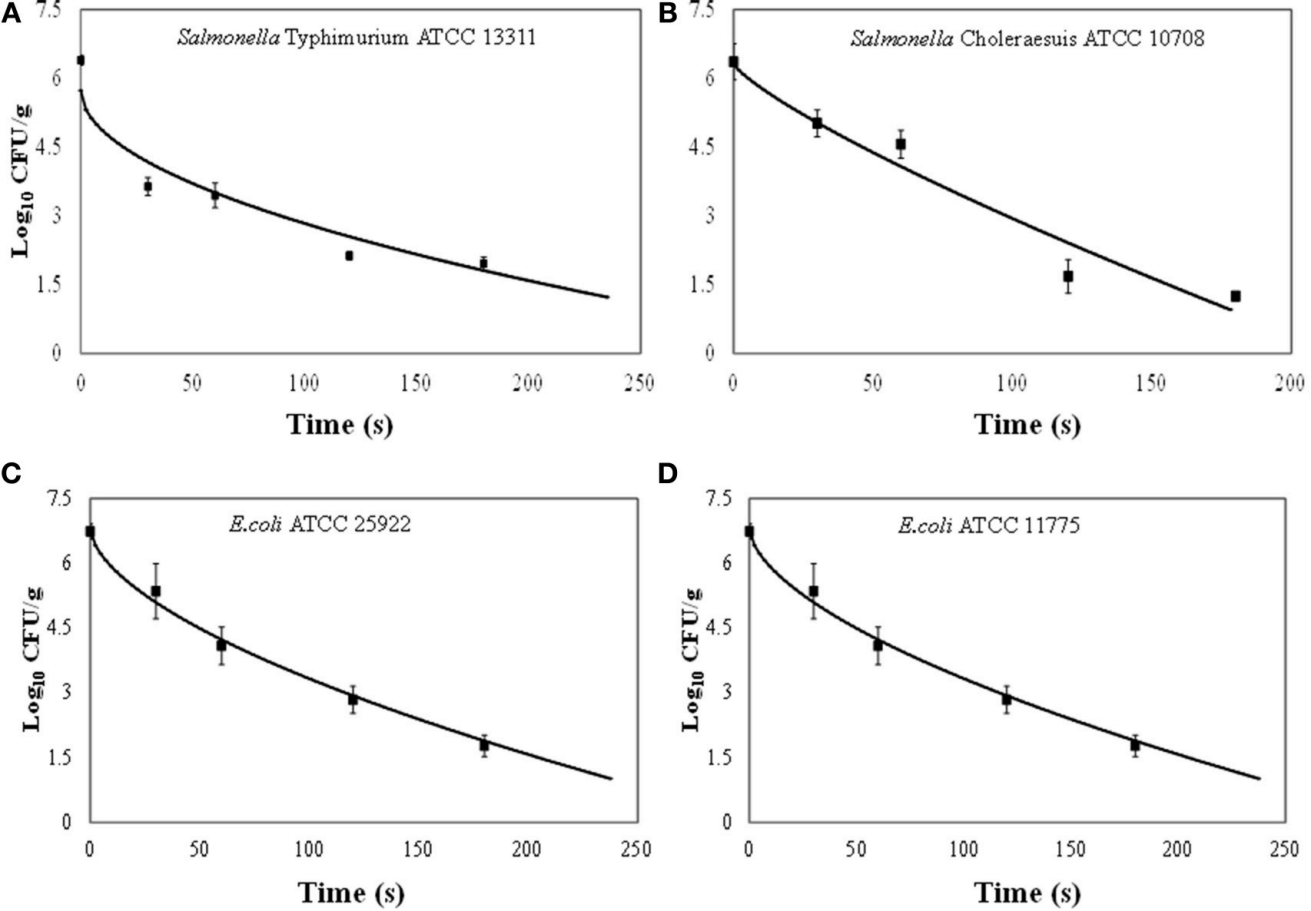
Effect of treatment time on the inactivation of *Salmonella* Typhimurium, ATCC 13311 **(A)**, *Salmonella* Choleraesuis, ATCC 10708 **(B)**, *Escherichia coli* ATCC 25922 **(C)**, *Escherichia coli* ATCC 11775 **(D)** on apples by the atmospheric cold plasma treatment using air as the plasma-forming gas. Error bars denote standard deviations.

The initial concentration (N_0_) of *Salmonella* or *E. coli* cells on each apple was approximately 10^7^ CFU/cm^2.^ Survival curves showed non-linear inactivation kinetics (Figures 3A,B). Generally, the reductions for both *E. coli* strains were not significantly different at the same exposure time intervals. *E. coli* populations were reduced by >1 log after 60 s. The concentration of *E. coli* significantly decreased (>5.0 log) at 180 and 240 s. *E. coli* ATCC 25922 and *E. coli* ATCC 11775 reductions ranged from 1.4 to 5.3 and 0.6 to 5.5 log_10_ CFU/cm^2^, respectively. The ACP inactivation of *E. coli* ATCC 25922 and *E. coli* ATCC 11775 on apples is presented in Figures 3C,D). Our results are in agreement of previous studies that demonstrated antimicrobial efficiency of cold plasma treatment on E. coli cells on apples. In an earlier study, *E. coli* O157:H7 populations on Golden Delicious apples were reduced by 3.5 and 3.0 log_10_ CFU/cm^2^, respectively after 180 s exposure at a flow rates of 30 or 40 ml/min (29). The application of one atmosphere uniform glow discharge plasma system (OAUGDP) has been reported to reduce *E. coli* O157:H7 on Red Delicious apples by 3 log_10_ CFU/cm2 after 120 s exposure (27). Ziuzina et al. (35) demonstrated that ACP reduced *E. coli* on strawberries by 1.2 and 1.6 log_10_ CFU/sample after 60 s and 120 s exposure, respectively, with significantly different reductions of 3.5 log_10_ CFU/cm^2^ after treatment for 300 s (*p* < 0.05). Recent studies have shown that plasma is a source of heat, UV irradiation, charged particles, reactive oxygen, and nitrogen based species (ROS and RNS, respectively) with a main role given to the species as disinfectants (20–23, 44–47). Our findings clearly demonstrated that increasing the treatment time caused an increased antimicrobial efficacy of ACP against the two strains of *E. coli*.

Inactivation of *Salmonella* Typhimurium and *Salmonella* Choleraesuis on apples is presented in Figures 3A,B. Inactivation of *Salmonella* strains showed a time-dependent reduction for all treatments. *Salmonella* Typhimurium and *Salmonella* Choleraesuis reductions ranged from 2.8 to 4.8 and 1.3 to 5.3 log_10_ CFU/cm^2^, respectively. Generally, *Salmonella* inactivation followed the same trend as for *E. coli* where reductions for both strains were not significantly (*p* < 0.05) different at the same exposure time intervals. After 30 s and 60 s exposure, *Salmonella* populations were reduced by >1 log. At higher treatment times (180–240 s), *Salmonella* Typhimurium and *Salmonella* Choleraesuis were reduced to 4.4 to 5.3 log_10_ CFU/cm^2^, respectively. *Salmonella* Typhimurium was slightly resistant to plasma as compared to other *E.Coli* and other *Salmonella* strain. The detection limit in the current study was 2 log_10_ CFU/apple piece.

Our findings suggest that *Salmonella* populations decreased as the treatment time increased. The relationship between the level of microbial log reduction and treatment time was observed to be non-linear (Figures 3A,B). Our results are in agreement of previous studies that demonstrated antimicrobial efficiency of cold plasma treatment on *E. coli* and *Salmonella* on apples. In an earlier study, *Salmonella* populations on Golden Delicious apples were reduced by 3.5 and 3.0 log_10_ CFU/cm^2^, respectively, after 180 s exopause at flow rates of 30 or 40 ml/min (29). The application of one atmosphere uniform glow discharge plasma system (OAUGDP) has been reported to reduce *Salmonella* on Red Delicious apples by 3 log_10_ CFU/cm^2^ after 120 s exposure (27). In the same study, correspondingly *Salmonella* reductions were 1.7 and 3.8 log_10_ CFU/sample after exposure for 120 and 300 s, respectively. Reduction of *E. coli* O157:H7, *Salmonella*, and *L. monocytogenes* have also been reported for apples and lettuce using gas plasma technology (48). Possible mechanism of inactivation may be reactive species produced in plasma which react with the amino-acid in proteins and further cause structural changes in proteins and results in destruction of the vegetative cells (49). In addition, OH, O, and O_3_ could break structural bonds in cell wall component pedtidoglycan, like C-O, C-N bonds, leading to cell wall destruction (50) and cell death. *Salmonella* and *E. coli* are Gram negative bacteria with a thinner outer membrane compared to the Gram positive *L. monocytogenes*. Clearly, the cell characteristics are critical factors for inactivation efficacy, but no clear trend is apparent from this study and highly complex interactions with the system, process, surface or medium may also impact on efficacy in combination with cell type.

### Modeling Inactivation Kinetics

Analysis of kinetics data showed that Weibull model was a good fit for the experimental data obtained (Table 1). Both bacteria studied were susceptible to plasma treatment, but experimental inactivation data and predicted parameters indicated that *Salmonella* Typhimurium ATCC 13311 was most resistant to plasma treatment than other microbes. It was observed that for 5 log reduction of *Salmonella* Typhimurium, a treatment time of 288.14 s is required. In contrast, *Salmonella* Choleraesuis required a treatment time of 163.39 s for 99.999% reduction.

Relative changes in bacterial concentration as a function of treatment time were fitted to Weibull model (*P* < 0.05) with coefficients of determination (Table 1) and a low RMSE. The *R*^2^ values of 0.92 and above (Table 1) show that the Weibull model was a good fit for the experimental data analyzed. *P*-values > 1 indicate the susceptibility of the remaining cells to the treatment. The parameter estimates were reported in terms of a rate constant. The t_5d_ (t_5d_–the time required for a 5 log_10_ reduction) for both *E. coli* and *Salmonella* strains is shown in Table 2. The treatment time varied between 288.14 and 163.39 s. *Salmonella* Typhimurium was the most resistant bacteria.

**Table 2:** Treatment time required for 5 log reduction (99.999%) in bacterial population.

**Microbe**	**t_**5d**_ (s)**
*Salmonella* Typhimurium ATCC 13311	288.14
*Salmonella* Choleraesuis ATCC 10708	163.39
*Escherichia coli* ATCC 25922	186.20
*Escherichia coli* ATCC 11775	178.22

*Data reported as means*.

**Figure 4:**
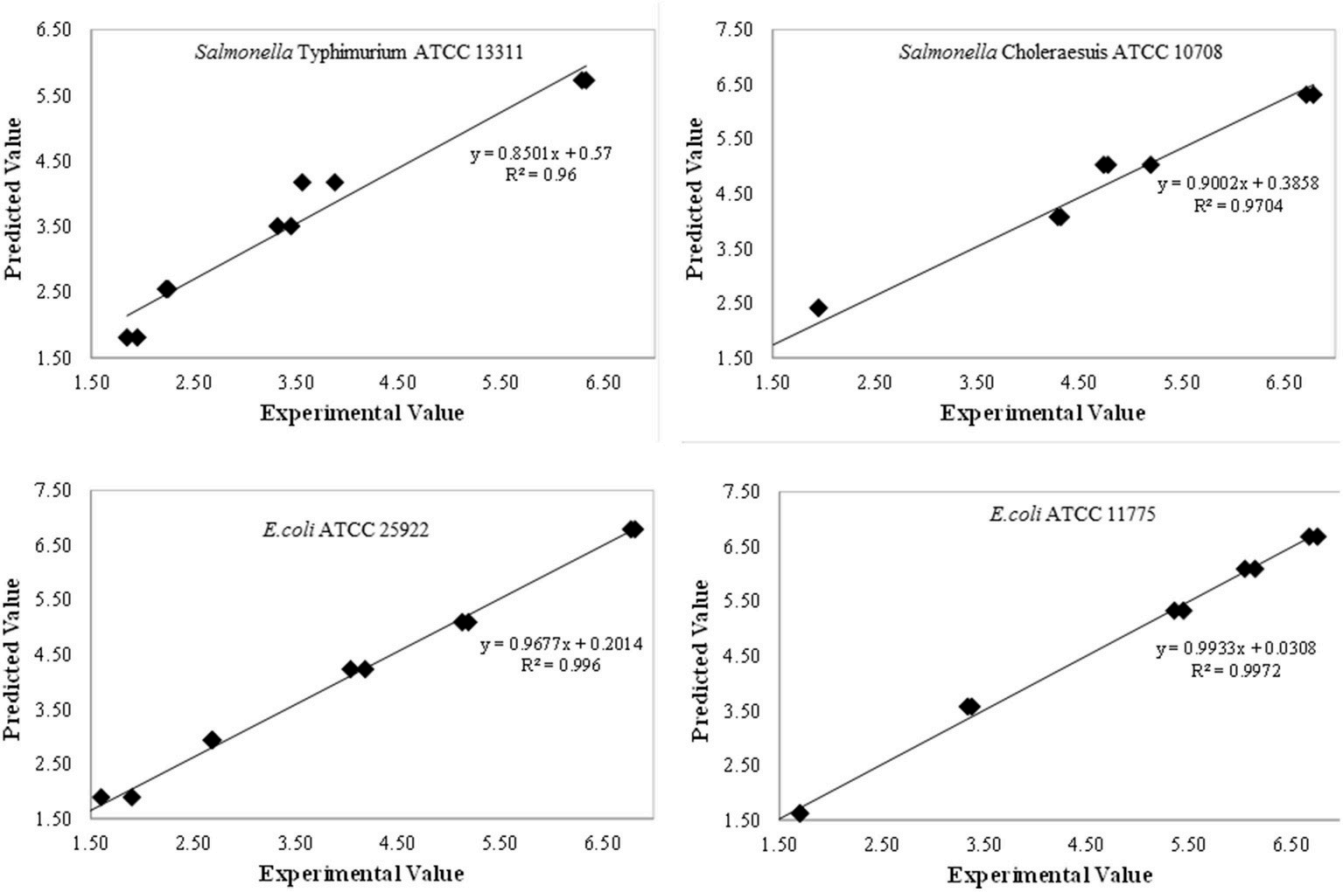
Model predictions and model efficacy.

The developed models for inactivation curves of pathogens describing the effect of plasma treatment on log reduction were validated using predictive modeling parameters. Independent set of experiments were conducted to validate the developed models. Predicted values of log reduction obtained using model equations were in good agreement with the experimental values. The experimental and predicted values were closely correlated with the experimental data as demonstrated by regression coefficient (R^2^) as shown in Figure 4. To confirm the adequacy of the fitted models, studentised residuals vs. run order were tested and the residuals were observed to be scattered randomly, suggesting that the variance of the original observations were constant for all responses. Further, the normality assumption was satisfied as the residual plot approximated to a straight line for all responses.

The average mean deviation (E) and multiple correlation coefficients (R^2^) were used to determine the fitting accuracy of data (51).

(3) $$\begin{array}{l} E\left(\%\right)=\frac{1}{n_{e}}\overset{n}{\underset{i=1}{\sum }}||\frac{V_{E}-V_{p}}{V_{E}}||\times 100 \end{array}$$

where, *n*_*e*_ is the number of experimental data, *V*_*E*_ is the experimental value and *V*_*P*_ is the predicted value.

The variation between the predicted and experimental log reduction values obtained for *E. coli* and *Salmonella* strains were within acceptable error range as depicted by average mean deviation (E%); therefore, the predictive performance of the established model may be considered acceptable (Tables 3, 4). It is indicated from the table that predicted values were in close agreement with the experimental values. The predicted values were found to be within the range of experimental values and were not significant at *p* < 0.05 using paired *t*-test.

**Table 3:** Average mean deviation for *Salmonella* strains.

**Experimental**	**Predicted**	**Error (%)**
***Salmonella*** **typhimurium**		
6.68	6.68	0.01
6.76	6.68	0.38
6.05	6.09	0.25
6.15	6.09	0.30
5.36	5.33	0.18
5.45	5.33	0.73
3.38	3.57	1.91
3.34	3.57	2.33
1.70	1.62	1.53
1.70	1.62	1.53
1.00	0.98	0.62
***Salmonella*** **choleraesuis 10708**		
6.79	6.31	2.34
6.72	6.31	2.01
4.78	5.03	1.76
4.74	5.03	2.05
5.20	5.03	1.08
4.29	4.08	1.65
4.32	4.08	1.87
1.95	2.41	7.89
1.95	2.41	7.89
1.20	0.91	8.06
1.20	0.91	8.06

**Table 4:** Average mean deviation for *E.coli* strains.

**Experimental**	**Predicted**	**Error (%)**
***Escherichia coli*** **ATCC 25922**		
6.54	6.79	1.28
6.94	6.79	0.71
4.78	5.09	2.19
4.58	4.23	2.53
4.04	4.23	1.59
3.15	2.94	2.27
2.69	2.94	3.04
1.60	1.89	6.05
1.60	1.89	6.05
1.00	0.98	0.62
1.00	0.98	0.62
***Escherichia coli*** **ATCC 11775**		
6.39	6.68	1.52
6.68	6.68	0.01
6.05	6.09	0.25
6.16	6.09	0.35
5.20	5.33	0.84
5.45	5.33	0.73
3.38	3.57	1.91
3.38	3.57	1.91
3.76	3.57	1.65
1.60	1.62	0.46
1.70	1.62	1.53

## Conclusions

Our results indicate that atmospheric cold plasma inactivated *E. coli* and *Salmonella* strains on the surface of Golden Delicious apples. Inactivation times for a ≈ 5 log cycle reduction ranged between 120 and 240 s. The inactivation depended significantly on treatment time and was well explained by the Weibull model. The results presented in the current study demonstrate the efficacy of ACP in activation of *E. coli* and *Salmonella* strains on apples. Overall, our results demonstrated the potential of atmospheric cold plasma as a means of improving the microbiological safety of fruits. Future studies are needed to address the feasibility for scale-up of this technology to pilot and commercial scales for decontamination pathogenic bacteria on fresh produce.

## Author Contributions

AK-N conducted the experiments and evaluated the results. SL conducted the experiments. AP conducted the kinetic modeling. SY validated and optimized the gas plasma system.

### Conflict of Interest Statement

The authors declare that the research was conducted in the absence of any commercial or financial relationships that could be construed as a potential conflict of interest.
